# A Mass Conservative Kalman Filter Algorithm for Computational Thermo-Fluid Dynamics

**DOI:** 10.3390/ma11112222

**Published:** 2018-11-08

**Authors:** Carolina Introini, Stefano Lorenzi, Antonio Cammi, Davide Baroli, Bernhard Peters, Stéphane Bordas

**Affiliations:** 1 Politecnico di Milano, Department of Energy, via La Masa 34, I-20156 Milano, Italy; carolina.introini@polimi.it (C.I.); stefano.lorenzi@polimi.it (S.L.); 2 Department of Computational Engineering Sciences, Faculty of Science, Engineering and Communication, University of Luxembourg, 6 Avenue de la Fonte, 4364 Esch-sur-Alzette, Luxembourg; davide.baroli@uni.lu (D.B.); bernhard.peters@uni.lu (B.P.); stephane.bordas@uni.lu (S.B.); 3 School of Engineering, Cardiff University, Queen’s Building, The Parade, Cardiff CF24 3AA, Wales, UK; 4 Department of Medical Research, China Medical University Hospital, China Medical University, Taichung 40402, Taiwan

**Keywords:** computational fluid-dynamics, OpenFOAM, Kalman filter, mass conservation, data assimilation, lid-driven cavity

## Abstract

This paper studies Kalman filtering applied to Reynolds-Averaged Navier–Stokes (RANS) equations for turbulent flow. The integration of the Kalman estimator is extended to an implicit segregated method and to the thermodynamic analysis of turbulent flow, adding a sub-stepping procedure that ensures mass conservation at each time step and the compatibility among the unknowns involved. The accuracy of the algorithm is verified with respect to the heated lid-driven cavity benchmark, incorporating also temperature observations, comparing the augmented prediction of the Kalman filter with the Computational Fluid-Dynamic solution found on a fine grid.

## 1. Introduction

Data assimilation is a process that allows handling experimental or real-time measurements inside the modeling framework. The dynamic data-driven method allows analysts to infer data from the Bayesian approach and analyze the correlation between the predicting model and the relevant knowledge from experimental measurements. These features open the possibility to learn from the many experimental datasets available in the industrial process in a non-deterministic setting. In addition, data assimilation can be used in combination with a simplified mathematical-physics modeling to obtain an accurate, thanks to the data-driven method, as well as fast running, thanks to the simplified modeling approach, representation of the phenomena to be studied [1] .

Among the wide range of possible applications, the focus of this study is the computational fluid-dynamics (CFD) with conjugate heat transfer. In several fields, the simulation-based CFD modeling is of paramount importance. For example, in nuclear applications, this modeling effort has the main purpose to assess the safety analysis of the reactor under normal and accidental conditions [2,3,4]. In the fluid-dynamics field, the challenging question is how to incorporate available data from fluid flow and temperature measurements into the available models, and how to take into account the discrepancy between the model prediction and the knowledge of the scenario coming from the data. In this sense, the use of data assimilation is a strong potential tool to increase the robustness and reliability of the analysis with a compatible computational burden. At the state of art, the integration of data assimilation in computational fluid dynamics is recently investigated in a number of different contexts, such as geosciences [5,6,7], biomedical simulations [8,9], and turbulence flow [10,11], to name a few.

When dealing with the modeling of complex systems, one of the main challenges is related to the error model and uncertainties that may origin from lack of knowledge of the underlying physics—because all the phenomena involved in the system are not known, or because some of these phenomena are too difficult to model—or from non-affordable computational burden to resolve adequately the physical model. For instance, in the analysis of turbulent flows, the last source of discrepancy occurs in the modeling of the turbulent flow itself, where different modeling approaches can be applied according to the level of accuracy required from the application field. Reynolds-Averaged Navier–Stokes (RANS) modeling, whether introducing several modeling approximations in the treatment of the fluid structure, is usually preferred in the engineering field with respect to more accurate but computationally expensive approaches, such as Large Eddy Simulation (LES) or Direct Numerical Simulation (DNS). RANS, and in general low fidelity approaches, are usually able to provide time-averaged quantities of the main variables of interest, being this level of accuracy sufficient in many engineering applications. On the other hand, the knowledge of the state of the system calculated with these low fidelity models can be enriched, combining real-time sensor data to obtain a better estimation of the variables of interest in the system and correcting the possible departure from the real value due to the model uncertainties.

In addition to the model discrepancies, the degree of uncertainty is also associated with the data acquisition. In particular, the observation is considered as a random process subjected to a density function selected a priori or a posteriori. Mathematically, the formulation of the data-driven method is equivalent to a Bayesian inverse problem in which the state of the system, governed by a set of partial differential equations, is identified sparsely by the data affected by uncertainty. In broad sense, fixing a realization of a random process, the inverse problem leads to the minimization of the following:

(1) $$\chi^{2}=\sum_{i=1}^{N}\frac{{\left(u_{i,obs}-u_{i,predict}\right)}^{2}}{\sigma_{i}^{2}}$$

where $\chi_{2}$ is the chi-squared statistics, $u_{obs}$ and $u_{predict}$ denote, respectively, the field observed and predicted, and $\sigma^{2}$ represents the uncertainty. Given the complexity of fluid dynamics problems, the open-box idea in data-driven approaches is used, instead of the black-box algorithm used in system identification introduced in [12]. In the former approach, the data driven scheme is incorporated in the modeling formulation [13] to handle the modeling constraints as the null divergence of the velocity field in incompressible flows or the consistency between pressure and velocity field imposed by the Navier–Stokes equations. This last constraint leads to mass conservation and positive semi-definiteness of Reynolds stresses, which are fundamental requirements to achieve a realistic prediction.

The data-driven algorithms can be divided into sequential and non-sequential ones. Among the latter, the variational approaches, such as 3D-VAR and 4D-VAR, are the most employed [14]. They are based on the adjoint operator and optimal control problem, where the misfit defines the functional constraints of the governing partial differential equations. These approaches allow a highly reliable simulation and a sensitive analysis from the adjoint analysis. However, their application to fluid dynamics investigation is effective only over long observation windows and the implementation of adjoint-based solvers is not available for many simulation tools. On the other hand, the sequential methods are based on the past observations of the system and from a mathematical point of view rely on Bayesian covariance and on the resolution of Riccati-type equations [15]. In particular, the covariance matrix is computed based on linear or pseudo-linear forward model or its approximation via a sampling method. Among these algorithms, our investigation is focused on the Kalman filter due to its straightforward integration in the segregate approaches usually employed in fluid dynamics solvers based on Finite Volume (FV) approximation. From the computational point of view, the main drawback of using a Kalman filter in such turbulence modeling is related to the assembling of the a priori error covariance and model covariance, due to the high number of degree of freedoms (the number of elements of the numerical grid) and thus the high computational cost.

In the present work, an approach that applies the sequential Kalman filter data-driven algorithm to the computational thermo-fluid dynamics modeling based on RANS assumption is proposed. With respect to the algorithm developed in [16], this work focuses on the heat transfer problem by implementing the data assimilation method for temperature observations. Heat transfer is of fundamental importance in any industrial problem. Unexpected temperature variations usually implies non-optimal operating conditions of the equipment, which may lead to faults and accidents. Real-time monitoring of temperature and its variations is a common practice, but the accurate simulation and prediction of heat transfer remains an open issue. Uncertainties in heat transfer modeling is for example related to the empirical correlations adopted for the prediction of convective heat transfer coefficients, whose validity and accuracy depend, among others, on the turbulent flow itself. The degree of turbulence greatly influences heat transfer, adding another layer of uncertainty. In addition to the treatment of temperature, the incorporation of Kalman filtering with the segregated method that combines the Pressure Implicit Split Operator (PISO) and Semi-Implicit Method for Pressure-Linked Equations, called PIMPLE, is investigated. With respect to PISO, already implemented in [16], the PIMPLE algorithm offers more robustness and efficiency when simulations with large time steps are of interest, as in some pseudo-transient cases. Being an implicit method, PIMPLE is less sensitive to numerical instability, and more suited for stiff problems, for which the use of an explicit method would require impractically small time steps to keep the solution stable.

It is worth noting that, usually, the acquisition of experimental data for velocity is not easy, whereas temperature measurements are more simple to obtain. A common practice in literature is to use DNS (Direct Numerical Simulation) solutions as placeholders for velocity experimental data, however as stated before such method is computationally not feasible for complex cases. Therefore, the ability of the developed integrated algorithm to improve the prediction of both quantities of interest while having access to only temperature experimental data is investigated. This represents quite a strong feature of the proposed approach.

The structure of the paper reads as follows. In Section 2, we describe the fundamental algorithms, namely, the Kalman filter and the segregated approach for the non-linear turbulence model. Section 3 is devoted to the proposed algorithm and how the physical properties are enforced in an open-box framework. In Section 4, the method is verified with respect to the benchmark case of the lid-driven cavity, before drawing conclusions in Section 5.

## 2. Numerical Background

The main objective of this work is to develop a CFD-based algorithm for thermo-fluid problems integrated with the sequential Kalman filter data-driven algorithm. Among all available CFD approaches for the Reynolds-Averaged Navier–Stokes equations, the segregated method, commonly used in the Finite Volume discretization framework, was chosen due to its popularity in the computational fluid-dynamics field, as well as its two-step structure, formed by state prediction and correction. This two-step framework is very reminiscent of the structure of the sequential Kalman filter, in which the state prediction, performed by the state model, is followed by an update performed by combining the prediction to the information obtained from the experimental measurements, suitably weighted. Due to this similarity, the integration of the sequential Kalman filter within the segregated method for CFD is natural. In the following, both the linear Kalman filter and the general framework of the segregated method are briefly introduced, without entering into details about their numerical properties. More information on the segregated methods and the Kalman filter can be found in [17,18], respectively.

### 2.1. The Kalman Filter

A Kalman filter is a set of Riccati-type equations which provide a recursive estimator based on a predictor-update procedure. This estimator is shown to be optimal in the sense that it minimizes, under some assumptions, the mean of the squared error, defined as the difference between the predicted state and the corrected one. One of such hypothesis is that the inferred distribution of the experimental data must be Gaussian, and that the numerical and observation models must be linear [19].

The aim of the Kalman filter is to estimate the state $u\in R^{N}$ of a given discrete-time system governed by the following linear equation:

(2) $$u_{n}=\underline{\underline{\Phi_{n}}}u_{n-1}+\underline{\underline{B_{n}}}c_{n}+w_{n},$$

where $u_{n}$ represents the a priori state estimate, with a measurement $z\in R^{M}$ governed by the following relation:

(3) $$z_{n}=\underline{\underline{H_{n}}}u_{n}+v_{n},$$

where $\underline{\underline{\Phi_{n}}}\in R^{N\times N}$ is the state transition matrix that describes the evolution of the system, $\underline{\underline{B_{n}}}\in R^{N\times L}$ represents the control matrix which relates the the control state vector $c_{n}\in R^{L}$ to the state vector, $w_{n}\in R^{N}$ contains the uncertainties associated with the model itself, $\underline{\underline{H_{n}}}:\left(n\Delta t\right)\in R^{M\times N}$ denotes the transformation matrix, needed to project the state variables in the space of observations, and the vector $v_{n}\in R^{M}$ represents the noise associated with the measurements.

By defining ${\hat{u}}_{n,n}^{*}$ as the a posteriori state estimate at time step *n* given the measurement $z_{n}$ and the knowledge of the state priori to step *n*, the a posteriori estimate error and its covariance can be defined as:

(4) $$\varepsilon_{n}=u_{n}-{\hat{u}}_{n,n}^{*};$$

(5) $$\underline{\underline{P_{n,n}}}=E\left[\varepsilon_{n}\varepsilon_{n}^{T}\right].$$

The strength of such an approach lies in the fact that even few measurements taken at a few time steps and at a few locations of the domain are enough to provide a state estimate which is better than the one obtained without measurements and to steer the numerical solution towards the true one. This means that the rank of $\underline{\underline{H_{n}}}$ can be much smaller than that of $\underline{\underline{\Phi_{n}}}$.

The a posteriori estimate ${\hat{u}}_{n,n}^{*}$ is computed as a linear combination of the a priori estimate $u_{n,n-1}$ and a weighted difference between the actual measurement and a measurement prediction:

(6) $${\hat{u}}_{n,n}^{*}=u_{n}+\underline{\underline{K_{n}}}\left(z_{n}-\underline{\underline{H_{n}}}u_{n}\right).$$

The difference $\underline{\underline{K_{n}}}\left(z_{n}-\underline{\underline{H_{n}}}u_{n}\right)$ is called the measure innovation, and reflects the discrepancy between the predicted measurement and the actual one. The matrix $\underline{\underline{K_{n}}}\in R^{N\times M}$ is called gain, and it minimizes the a posteriori error.

Since the a priori distribution of the error models is not known, it is assumed that all the inferred distributions are Gaussian, statistically independent of each other and not temporally correlated. For the observation, a Gaussian distribution is also assumed, due to its relation with the white noise commonly associated with measurements [18,20]. For this reason, the uncertainty associated both to the model and to the observation is taken as described by random variables with Gaussian distribution:

(7) $$p\left(w_{n}\right)∼N\left(0,Q_{n}\right);$$

(8) $$p\left(v_{n}\right)∼N\left(0,R_{n}\right).$$

The equations for the predictor and update step are as follows:

- **Predictor step**
  (9) $${\hat{u}}_{n}=\underline{\underline{\Phi_{n}}}u_{n-1}+\underline{\underline{B_{n}}}c_{n-1}+w_{n};$$
  (10) $$\underline{\underline{P_{n,n-1}}}=\underline{\underline{\Phi_{n}}}\underline{\underline{P_{n-1,n-1}}}\underline{\underline{\Phi_{n}^{T}}}+Q_{n}.$$
- **Update step**
  (11) $$\underline{\underline{K_{n}}}=\underline{\underline{P_{n,n-1}}}\underline{\underline{H_{n}^{T}}}{\left(R_{n}+\underline{\underline{H_{n}}}\underline{\underline{P_{n,n-1}}}\underline{\underline{H_{n}^{T}}}\right)}^{-1};$$
  (12) $${\hat{u}}_{n,n}^{*}={\hat{u}}_{n,n-1}+\underline{\underline{K_{n}}}\left(z_{n}-\underline{\underline{H_{n}}}{\hat{u}}_{n,n-1}\right);$$
  (13) $$\underline{\underline{P_{n,n}}}=\left(\underline{\underline{I}}-\underline{\underline{K_{n}}}\underline{\underline{H_{n}}}\right)\underline{\underline{P_{n,n-1}}}.$$

Given an initial guess ${\hat{u}}_{0,0}^{*}$ and an initial value for the covariance matrix $\underline{\underline{P_{0,0}}}$, the predictor step equations allow obtaining the a priori state estimate and covariance matrix estimate. Once the observation is available, the update step starts with the evaluation of the Kalman gain, followed by the a posteriori state estimate by incorporating the measurement. The final step is to obtain the a posteriori error covariance estimate. The process is then repeated with the previous a posteriori estimate used as initial guess for the new a priori one.

The Kalman filter can therefore be seen as a Bayesian approach, where the *best estimate* is interpreted in the sense of the Maximum A-Posteriori (MAP). The MAP estimate of a random variable u is defined as the estimation ${\hat{u}}^{*}$ which, given the observation vector Z and the knowledge of how both measurement and model approximated prediction are flawed, maximizes p(u|Z). The Bayes theorem allows inferring the a posteriori distribution $p(u_{n}|Z_{n})$ starting from the vector of past measurements:

(14) $$p\left(u_{n}|z_{n}\right)=\frac{p(u_{n},Z_{n})}{p(Z_{n})}=\frac{p(u_{n},z_{n},u_{n-1})}{p(z_{n},Z_{n})}$$

For the Kalman filter, the a priori distribution is represented by the model estimation, the likelihood by the measurement, and the a posteriori distribution by the MAP estimate defined above. More detailed descriptions of the Kalman filter procedure can be found in [21,22].

### 2.2. The Segregated Method

The segregated approach for the resolution of the Navier–Stokes equations, along with the energy equation, is based on an iterative procedure that involves the decoupling between velocity, pressure and temperature equations. Since the constitutive law of Newtonian incompressible fluid is chosen, the evolution of the velocity field u, the normalized pressure *p*, defined as the absolute pressure over the density of the medium, and temperature *T* can be described as:

$$\left\{ \begin{array}{cc} \nabla \cdot u=0; & \;(15)\\ \frac{\partial u}{\partial t}+\nabla \cdot \left(uu\right)-\nabla \cdot \left(\nu \nabla u\right)=-\nabla p-g\beta \left(T-T_{ref}\right); & \;(16)\\ \frac{\partial T}{\partial t}+\nabla \cdot \left(uT\right)=k\Delta T, & \;(17) \end{array}\right.$$

where u represents the velocity vector, ν is the kinematic viscosity of the fluid, *p* is the normalized pressure, g is the gravity acceleration, β is the thermal expansion coefficient of the fluid, *T* is the fluid temperature, $T_{ref}$ is a reference temperature, and *k* is the thermal diffusivity of the fluid.

Two of the most well-known segregated algorithms for the approximation of the time-dependent Navier–Stokes equations are the PISO (Pressure Implicit with Splitting of Operator) and PIMPLE (Merged PISO-SIMPLE, where SIMPLE stands for Semi-Implicit Method for Pressure Linked Equations). Both methods follow the same procedure for the discretization, but, with respect to PISO, the PIMPLE algorithm is characterized by an additional inner iteration loop which resolves the non linearity of the Navier–Stokes equations and the energy equation with Picard iterations. This inner loop makes the PIMPLE algorithm an implicit method, whereas the PISO algorithm is a semi-implicit method, since the velocity correction is solved explicitly. The iteration process of the PIMPLE algorithm allows for simulations with under-relaxation of the solution to improve stability, and adaptivity of the time step as the simulation progresses. As stated in Section 1, the PIMPLE method is less sensitive to numerical instability and more suited for stiff problems.

In general, all segregated methods presents a two-step structure, composed by a predictor and a corrector step. The equations for these two steps are summarized as follows:

- **Predictor step**
  (18) $$a_{p}u_{p}=-\sum \left(a_{k}u_{k}\right)+\underline{\underline{\Psi_{n-1}\left(u_{n-1}\right)}}-\nabla p_{n-1}=\underline{\underline{\Psi }}\left(u\right)-\nabla p_{n-1};$$
  (19) $$a_{T,p}T_{p}=-\sum \left(a_{T,k}T_{k}\right)+\underline{\underline{\Psi_{T,n-1}}}\left(T_{n-1}\right)=\underline{\underline{\Psi_{T}}}\left(T\right);$$
  $$\rho_{p}=1-\beta_{p}\left(T_{p}-T_{ref}\right).$$
- **Corrector step**
  (20) $$\nabla \cdot u=\sum (S\times u_{f})=0;$$
  (21) $$u_{f}={\left(\frac{\underline{\underline{\Psi }}\left(u\right)}{a_{p}}\right)}_{f}-\left(\frac{\nabla p}{a_{p}}\right);$$
  (22) $$\nabla \cdot \left(\frac{\nabla p}{a_{p}}\right)=\nabla \cdot \left(\frac{\underline{\underline{\Psi }}(u}{a_{p}}\right)=\sum \left(S\times {\left(\frac{\underline{\underline{\Psi }}(u}{a_{p}}\right)}_{f}\right).$$

In the above, the subscript *P* represents the discretized field in the considered grid element, *N* indicates its neighbours, and $a_{p}$ and $a_{n}$ are the coefficients that result from the discretization procedure. In the case of PISO, the subscript n−1 refers to the previous time step, whereas, in the case of PIMPLE, it refers to the previous iteration within the same time step. The non-linearity of the momentum equation is contained in the term $a_{p}$ and the operator $\underline{\underline{\Psi }}$. The subscript *f* stands for an interpolation on the face centres of the mesh elements and *S* is the corresponding face area.

The predictor of the velocity and temperature fields is obtained by approximating the momentum Equation (16) and energy Equation (17), respectively. In the former, the non-linear advection term is simplified by linearization of the new system state around the one at the previous time step (for PISO) or the one at the previous inner iteration (for PIMPLE), which represents the right hand side of the equation. This approximation relies on the hypothesis of small values of the time step Δt, and the error of such approximation proportionally propagates to $\Delta t^{2}$ [17].

Equations (18) and (19) are solved through iterative techniques. However, usually the predicted velocity field does not comply with the zero-divergence condition. This requirement is expressed by Equations (20) and (21). Combining these two equations, a Poisson equation for pressure, which expresses the zero-divergence condition, is obtained [23]. This pressure field does not necessarily satisfy Equation (18), but it is used to correct the predicted velocity field. This predictor–corrector loop continues until a prescribed convergence criterion is satisfied. In the case of PIMPLE, multiple cycling of the predictor–corrector loop over the same time step are performed using the last iteration final value as initial guess for the next one r to improve the convergence of the solution. While the Navier–Stokes equations are non-linear, the segregated method includes the non-linearity into a linear system resolution. This feature is essential for the implementation of the Kalman filter within a segregated framework, as shown in Section 3.

## 3. Method: The Integrated Algorithm

**Figure 1:**
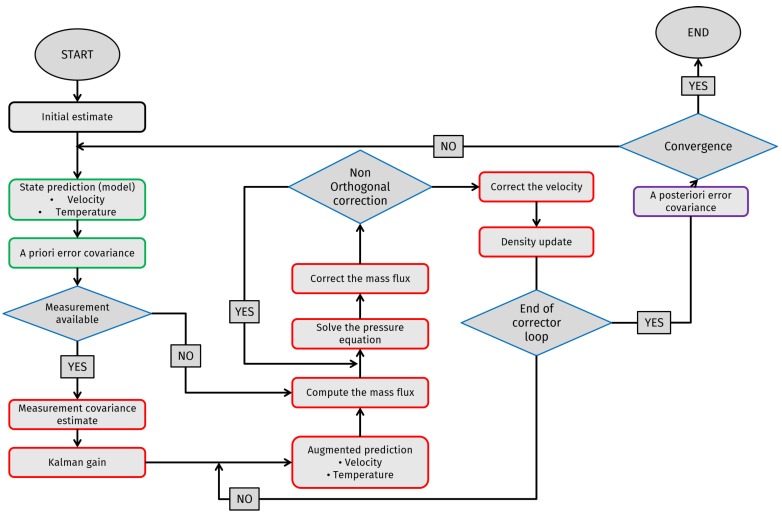
Flow chart of the integrated algorithm. The blocks with blue borders represent the prediction step, the ones with red borders are the corrector step, and the purple borders highlight the regularization step.

The present algorithm integrates the discrete Kalman filter introduced in Section 2.1 and the segregated methods presented in Section 2.2 to obtain a divergence-free augmented prediction. The approach used for the development of the integrated code is extracted from [16], with the extension of temperature and the implementation of the Kalman filter within the PIMPLE segregated method. In general, this integrated algorithm can be divided into three steps, namely a predictor, a corrector and a regularization step. The first two reflect the predictor–corrector structure of the segregated methods, whereas the latter represents the update step in the discrete Kalman filter algorithm. A flow chart outlining the steps of the integrated algorithm is presented in Figure 1.

At the beginning of the time step *n*, Equations (23) and (24) are used to estimate the system velocity and temperature given the ones at the previous time step (or the previous iteration, for PIMPLE). Regardless of the presence of the observation, the error covariance matrix is evaluated a priori by Equations (25) and (26), given the state transition matrix at the current time step. The model uncertainty introduced by the matrix $Q_{n}$ is propagated using the correlation between state variables provided by $\Phi_{n}$ and $\Phi_{T,n}$, and this information is stored in the error covariance matrices $P_{n,n-1}$ and $P_{T,n,n-1}$. The equations are as follow:

- **Predictor step**
  (23) $$a_{n,p}{\hat{u}}_{n,n-1}=\underline{\underline{\Phi_{n}}}\left({\hat{u}}_{n-1,n-1}\right)-\nabla p_{n-1,n-1};$$
  (24) $$a_{n,T,p}{\hat{T}}_{n,n-1}=\underline{\underline{\Phi_{T,n}}}\left({\hat{T}}_{n-1,n-1}\right);$$
  (25) $$\underline{\underline{P_{n,n-1}}}=\underline{\underline{\Phi_{n}}}\underline{\underline{P_{n-1,n-1}}}\underline{\underline{\Phi_{n}^{T}}}+Q_{n};$$
  (26) $$\underline{\underline{P_{T,n,n-1}}}=\underline{\underline{\Phi_{T,n}}}\underline{\underline{P_{T,n-1,n-1}}}\underline{\underline{\Phi_{T,n}^{T}}}+Q_{n}.$$

In the above, ${\hat{u}}_{n,n-1}$ and ${\hat{T}}_{n,n-1}$ are, respectively, the predicted system velocity and temperature given the ones at the previous time step; $\Phi_{n}$ and $\Phi_{T,n}$ are the state transition matrices for velocity and temperature; ${\hat{u}}_{n-1,n-1}$, $p_{n-1,n-1}$ and ${\hat{T}}_{n-1,n-1}$ are, respectively, the system velocity, pressure and temperature at the previous time step; $P_{n,n-1}$ and $P_{T,n,n-1}$ are the a priori error covariance matrices for velocity and temperature; $P_{n-1,n-1}$ and $P_{T,n-1,n-1}$ are the ones at the previous time step; and $Q_{n}$ is the model uncertainty matrix.

If the experimental measurement for the current time step is not available, the corrector step of the chosen segregated method is applied to the velocity prediction in order to obtain a divergence-free solution. Otherwise, the Kalman gains for velocity and temperature are evaluated, and the Poisson equation is used to impose the zero-divergence condition for the *augmented* prediction. In the case of the PIMPLE method, the correction for the velocity prediction with the observation is applied only in the last iteration of the predictor–corrector loop, that is, the one without under-relaxation.

- **Kalman gain evaluation**
  (27) $$\underline{\underline{S_{n}}}=R_{n}+\underline{\underline{H_{n}}}\underline{\underline{P_{n,n-1}}}\underline{\underline{H_{n}^{T}}};$$
  (28) $$\underline{\underline{S_{T,n}}}=R_{T,n}+\underline{\underline{H_{T,n}}}\underline{\underline{P_{T,n,n-1}}}\underline{\underline{H_{T,n}^{T}}};$$
  (29) $$\underline{\underline{K_{n}}}=\underline{\underline{P_{n,n-1}}}\underline{\underline{H_{n}^{T}}}\underline{\underline{S_{n}^{-1}}};$$
  (30) $$\underline{\underline{K_{T,n}}}=\underline{\underline{P_{T,n,n-1}}}\underline{\underline{H_{T,n}^{T}}}\underline{\underline{S_{T,n}^{-1}}}.$$

In the above, $S_{n}$ and $S_{T,n}$ are the measurement covariance matrices for velocity and temperature, respectively; $R_{n}$ and $R_{T,n}$ are the uncertainties associated with the experimental observation of velocity and temperature; $K_{n}$ and $K_{T,n}$ are the Kalman gain matrices; and $H_{n}$ and $H_{T,n}$ are the transformation matrices that map the state variables onto the observation matrix. In general, these matrices are defined as: $H_{i,j}=\left\{\begin{array}{cc} 1 & \mathrm{if}\;i=j\mathrm{and}x_{i}\;\mathrm{is}\;a\;\mathrm{location}\\ 0 & \mathrm{otherwise} \end{array}\right.$

- **Corrector step**
  (31) $${\hat{u}}_{n,n}^{*}={\hat{u}}_{n,n-1}+\underline{\underline{K_{n}}}\left(z_{n}-\underline{\underline{H_{n}}}{\hat{u}}_{n,n-1}\right)=\frac{\underline{\underline{\Psi_{n}}}\left({\hat{u}}_{n,n-1}\right)}{a_{n,p}}-\frac{\nabla p_{n}}{a_{n,p}}+\underline{\underline{F_{n}}};$$
  (32) $$\nabla \cdot {\left(\frac{\nabla p_{n}}{a_{n,p}}\right)}_{f}=\sum_{f}{\left(S\times \left(\frac{\underline{\underline{\Psi_{n}}}\left({\hat{u}}_{n,n-1}\right)}{a_{n,p}}+\underline{\underline{F_{n}}}\right)\right)}_{f};$$
  (33) $${\hat{u}}_{n,n}=\frac{\underline{\underline{\Psi_{n}}}\left({\hat{u}}_{n,n}^{*}\right)}{a_{n,p}}-\frac{\nabla p_{n}}{a_{n,p}}.$$

In the above, ${\hat{u}}_{n,n}^{*}$ is the augmented velocity prediction, $z_{n}$ is the observation, $p_{n}$ is the system pressure computed at the current time step, ${\hat{u}}_{n,n}$ is the divergence-free augmented velocity, used as source term for the next iteration, and $\underline{\underline{F_{n}}}=\underline{\underline{K_{n}}}\left(z_{n}-\underline{\underline{H_{n}}}{\hat{u}}_{n,n-1}\right)$ is the innovation term. The resulting algorithm guarantees that the predicted velocity ${\hat{u}}_{n,n}$ always respect the zero-divergence constraint, while this condition is observed for the velocity field ${\hat{u}}_{n,n-1}$ derived from the model only if $\nabla \underline{\underline{F_{n}}}=0$. With respect to temperature, since no corrector loop is applied on its equation, the augmented prediction is evaluated and used to compute the system density using the Boussinesq approximation.

- **Corrector step (temperature)**
  (34) $${\hat{T}}_{n,n}^{*}={\hat{T}}_{n,n-1}+\underline{\underline{K_{T,n}}}\left(z_{T,n}-\underline{\underline{H_{T,n}}}{\hat{T}}_{n,n-1}\right);$$
  (35) $$\rho_{n}=1-\beta \left({\hat{T}}_{n,n}^{*}-T_{ref}\right).$$

In the above, ${\hat{T}}_{n,n}^{*}$ is the augmented temperature prediction, $z_{T,n}$ is the temperature experimental observation, $\rho_{n}$ is the fluid density, β is the fluid thermal expansion coefficient, taken as constant in time, and $T_{ref}$ is a reference temperature.

The regularization step is performed only when observations are available. In this step, the error covariance matrices for the quantities of interest are updated, thus obtaining the a posteriori error covariance matrices.

- **Regularization step**
  (36) $$\underline{\underline{P_{n,n}}}=\left(\underline{\underline{I}}-\underline{\underline{K_{n}}}\underline{\underline{H_{n}}}\right)\underline{\underline{P_{n,n-1}}};$$
  (37) $$\underline{\underline{P_{T,n,n}}}=\left(\underline{\underline{I}}-\underline{\underline{K_{T,n}}}\underline{\underline{H_{T,n}}}\right)\underline{\underline{P_{T,n,n-1}}}.$$

### Limitations and Assumptions

The implementation of the Kalman filter within the segregated algorithm is quite straightforward, due to the similarities between the two-step structure of the two algorithms and the linear nature of the latter. However, the use of the developed method can be problematic when dealing with large systems such as those needed for the solution of turbulent flows. The main reasons are:

- The derivation of the state transition matrix $\underline{\underline{\Phi_{n}}}$ from the operator $\underline{\underline{\Psi_{n}}}$
- The evaluation of the matrix $Q_{n}$

Whereas the state transition matrix can be obtained from the operator $\underline{\underline{\Psi }}$, its derivation includes a matrix inversion, and, considering that this operator varies in time, the computational requirements for a direct evaluation of the matrix $\underline{\underline{\Phi_{n}}}$ are not acceptable. However, thermo-fluid mechanics problems exhibit diagonal-dominant state matrices [24], especially when dealing with turbulent configurations due to the small time scales that have to be imposed to capture the dynamics of the flow. In addition, most discretization schemes for the Navier–Stokes equations produce diagonally dominant matrices in order to guarantee the stability and convergence of the iterative solver [23]. Thus, many of the off-diagonal terms of the state matrix $\underline{\underline{\Phi }}$ are zero, and, according to Meldi [16], this matrix can be approximated as:

$$\left\{ \begin{array}{cc} \underline{\underline{\Phi_{n}}}=\frac{1}{a_{p,n}}\underline{\underline{\Psi_{n}}}\left({\hat{u}}_{n-1}\right)=\frac{1}{a_{n,p}\delta t}\underline{\underline{I}}; & \;(38)\\ \underline{\underline{\Phi_{T,n}}}=\frac{1}{a_{T,p,n}}\underline{\underline{\Psi_{T,n}}}\left({\hat{T}}_{n-1}\right)=\frac{1}{a_{T,n,p}\delta t}\underline{\underline{I}}. & \;(39) \end{array}\right.$$

This approximation, made in [16] for velocity and here extended also for temperature, implies that, if the matrix $\underline{\underline{P}}$ is initially diagonal, and Q and R are diagonal during the simulation, $\underline{\underline{P}}$ and $\underline{\underline{K}}$ are diagonal as well and, therefore, they are easy to manipulate without an excessive computational effort.

Overall, the structure of the matrix $Q_{n}$ is difficult to predict. Ideally, since this quantity represents the level of confidence associated with the numerical simulation, it should be derived accounting for model results. Its structure was investigated in detail in [16]. The most important conclusion found are that the optimized value for this quantity is directly linked with the truncation error of the numerical scheme multiplied by Δt, and that the level of confidence in the numerical model is related with the discretization error. Taking into account also the numerical errors due to turbulence modeling, the elements of the matrix $Q_{n}$ are locally calculated for the mesh element *i* as:

(40) $$Q_{i}=C\left(1+\frac{\nu_{T,i}}{\nu }\Delta t^{ot}\Delta x_{i}^{os}\right);$$

(41) $$Q_{T,i}=C\left(1+\frac{\alpha_{T,i}}{k}\Delta t^{ot}\Delta x_{i}^{os}\right).$$

where C∈[0,1] is a constant that indicates the end-user subjective level of confidence on the numerical model, the superscripts os and ot are related with the order of the numerical schemes used for time and space discretization, $\nu_{T}$ represents the turbulence scale viscosity introduced by the model, ν is the fluid kinematic viscosity, $\alpha_{T}$ is the turbulent thermal diffusivity, and *k* is the thermal diffusivity of the fluid. This form for *Q*, proposed by Meldi [16] for velocity and here extended also for temperature, represents a reasonable estimation of the uncertainty associated with the model, which is a priori unknown. This form of the matrix **Q** is optimized for the test case discussed in Section 4.

## 4. Results and Discussion

In this section, the developed algorithm is tested against the classic benchmark of the 2D lid-driven cavity [25]. Verification is performed by comparing the results obtained by the application of the developed algorithm with the ones obtained by application of the standard segregated method on a very fine numerical grid. In this test, the integrated algorithm is used along with numeric observations taken from a fine grid, although a future application could be its use along with real observations coming from an experimental campaign. It is important to stress that the 2D geometry is adopted only for the sake of simplicity, and that the algorithm can be easily extended to the 3D case. The performance of the tested approach is evaluated by means of the $L^{2}$ normalized error, computed as follows:

(42) $$\varepsilon_{L2}=\frac{\left|\right|q_{CFD}-q_{Kalman}\left|\right|}{\left|\right|q_{CFD}\left|\right|},$$

where $q_{CFD}$ represents the solution as computed by the standard algorithm on a fine grid (a so-called high-order solution) and $q_{Kalman}$ is the solution as evaluated by the developed algorithm (the so-called low-order solution). In addition, the average Kalman gain is used as parameter to evaluate the performance of the filter, as well as the normalized misfit between measurement and time solution:

(43) $$\mathrm{Misfit}=\max\left(\frac{q_{obs}-q_{Kalman}}{q_{obs}}\right).$$

As an additional figure of merit, the *chi-square* $\chi^{2}$ is also used [26]. The $\chi^{2}$ merit function is a maximum likelihood function, typically used as a criterion to fit a set of model parameters to a model process known as *least square fitting*. As the Kalman filter is a *recursive least square* filter, the overall $\chi^{2}$ is used as an additional figure of merit:

(44) $$\chi^{2}=\sum_{i=1}^{N}{\left(\frac{q_{i,obs}-q_{i,Kalman}}{\sigma_{i}}\right)}^{2}$$

where $\sigma_{i}$ is the variance associated with the measured state.

### 4.1. 2D Lid-Driven Cavity

In the lid-driven cavity, the motion of the fluid is generated by the uniform movement of the lid of the cavity. Due to the simple geometrical shape, the well-defined boundary conditions and the very large database of numerical results at different Reynolds numbers, this test is considered a benchmark test case at the state of the art.

**Figure 2:**
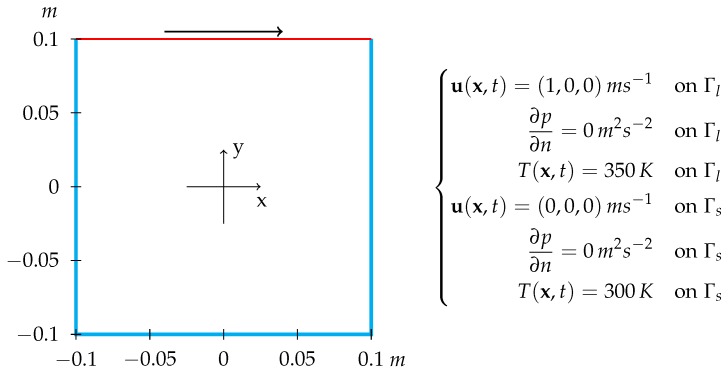
Domain of the 2-D lid driven cavity and imposed boundary conditions for velocity, pressure and temperature. The blue boundaries indicates the stationary walls, whereas the red one represents the lid moving in the positive x-direction. Dirichlet condition are imposed for velocity and temperature and a Neumann one is used for pressure. In the case without heat transfer, a Neumann condition is imposed also for temperature.

The motion of a viscous, Newtonian fluid such as water in an open cavity can be represented by the velocity, pressure and eventually temperature fields b=(u,p,T), whose behaviors are described with the two-dimensional, unsteady, incompressible Navier–Stokes equations, along with the energy equation written in temperature form. The strong form of such model reads:

$\mathrm{find}\;\left(u,p,T\right)\in H^{1}\left(\Omega \right)\times L^{2}\left(\Omega \right)\times H^{1}\left(\Omega \right)\;\mathrm{with}\;\Omega \in R^{d}\;(d\;=\;\mathrm{dimension}\;\mathrm{of}\;\mathrm{the}\;\mathrm{domain})\;\mathrm{such}\;\mathrm{that}\text{:}$

$$\left\{ \begin{array}{cc} \nabla \cdot u=0; & \;(45)\\ \frac{\partial u}{\partial t}+\left(u\cdot \nabla \right)u-\frac{1}{Re}\nabla^{2}u+\nabla p+g\beta \left(T-T_{ref}\right)=0; & \;(46)\\ \frac{\partial T}{\partial t}+u\cdot \nabla T=k_{eff}\Delta T, & \;(47) \end{array}\right.$$

where u denotes the velocity field, *p* represents the pressure, g is the gravity acceleration, *T* is the temperature field, $T_{ref}$ an arbitrary reference temperature, $k_{eff}$ is the medium effective thermal diffusivity, and Re indicates the Reynolds number, defined as follows:

$$Re=\frac{L_{ref}U_{0}}{\nu },$$

where $L_{ref}$ is the reference length, corresponding in this case to the dimension of the lid moving direction, $U_{0}$ is the modulus of the free-stream velocity and ν is the kinematic viscosity of the flow. For the considered case, this parameter is equal to $10^{5}$. Indicating the boundary of Ω with $\Gamma_{s}$ for the stationary walls and $\Gamma_{l}$ for the moving lid, a sketch of the lid-driven cavity geometry is shown in Figure 2.

**Figure 3:**
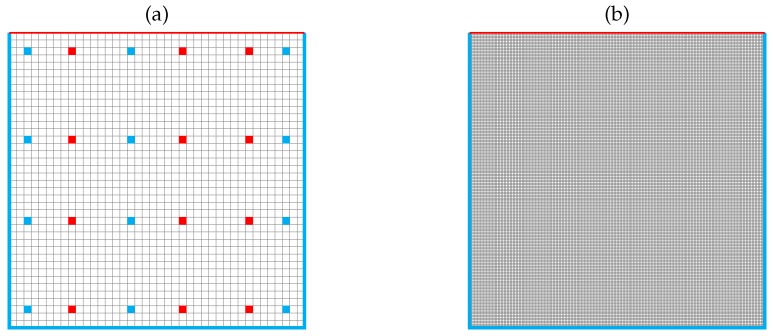
Numerical grids employed for the test case (not in scale). (**a**) medium-refined grid with 3600 elements and space discretization 0.002 m, used for the integrated algorithm. The blue and red dots represent, respectively, the locations where the artificial observation for velocity and temperature are taken. (**b**) High-refined grid with 100,000 elements and space discretization 0.00001 m, used to evaluate the reference CFD solution and to extract the synthetic data used as observation.

The computational domain is represented by a squared 2D cavity with a side of 0.1 m. Whereas the developed algorithm was applied to a grid with medium refinement, a fine one was used for the standard segregated method, to produce synthetic observations, and for comparison, as shown in Figure 3.

As experimental data for the filter, synthetic measurements are employed. These are taken from a CFD simulation performed on a very fine grid (100,000 elements) and perturbed with a known noise to obtain very accurate results. Since in most cases experimental data are provided only in sparse positions within the domain, this synthetic observation is taken only in a reduced number of domain cells, as shown in Figure 3. It must be noted how the observation positions are different for the two quantities of interest to simulate the case of real experimental data. Thus, the synthetic measurements of velocity and temperature are not strictly related one with the other, because the implementation of the Kalman filter treats them as two separate quantities. The random uncertainty associated with real measurements is assumed to follow a normalized Gaussian distribution, to model the white noise. Therefore, the experimental observations are represented by the input data (namely, the CFD simulation performed on a very fine grid) in sparse locations contaminated with randomly generated white noise.

### 4.2. Test Results

The performed test cases are presented by increasing complexity of the underlying problem. In all of them, the main focus is on numerical analysis to highlight the properties of the developed algorithms. The three cases considered are summarized in Table 1.

In all cases, the simulation is run until convergence is reached. As convergence criterion, the value of the initial residuals, being the normalized difference between the solution at two consecutive time steps, is taken. The tolerance of the iterative algorithm is fixed to $10^{-6}$, and when the initial residuals value falls below this threshold, the solution is considered converged, as it is not changing in a significant way.

#### 4.2.1. Kalman-PISO Algorithm

**Figure 4:**
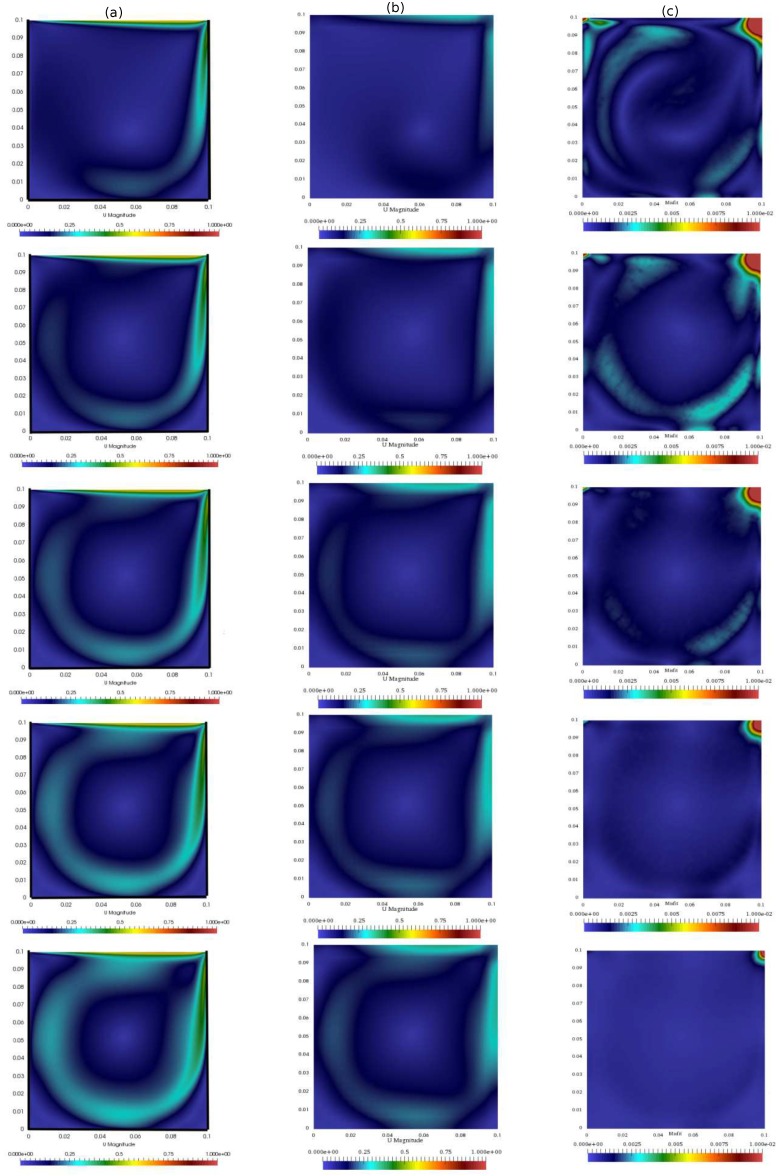
Contours of the velocity of the coupled algorithm with: the Kalman filter (**a**); the standard PISO algorithm (**b**); and the difference between the time solutions of the filter and of the standard algorithm computed on the finest mesh (**c**)

In the first test, the segregated PISO algorithm is coupled with the standard Kalman filter. The simulation is performed until 100 s with a constant time step of $5\cdot 10^{-3}\;$s. Figure 4 shows the velocity of the standard algorithm and of the integrated algorithm (evaluated on the same grid) at different times, along with the difference between the left solution and the reference one computed on the fine grid. As expected, this difference decreases in the overall domain except in the top right corner of the square cavity. Although in the present work the location of the observation was taken arbitrarily, the behavior of the misfit shown here can be used to a posteriori optimize the sensor’s location.

**Figure 5:**
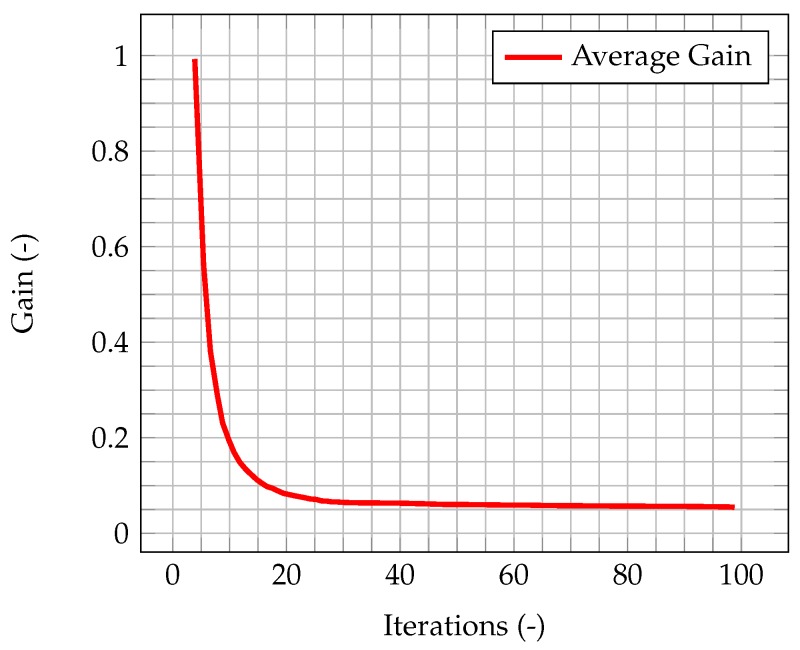
Time evolution of the average Kalman gain for velocity within the domain, highlighting its exponential-like behavior and its stationary value once convergence is reached.

In Figure 5, the time evolution of the average Kalman gain is shown. It can be seen how the mean value of the gain quickly decreases from 1, meaning that the experimental measurements weights more with respect to the model when computing the new system state, to a value very close to zero, meaning that the model weights more with respect to the experimental data.

**Figure 6:**
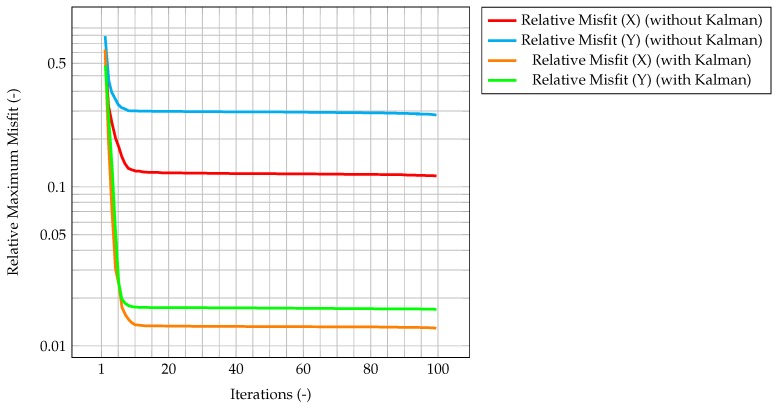
Time evolution of the maximum normalized misfit between observation and CFD solution. This misfit is computed as the difference between the observations (cleaned from the noise) and the CFD solution computed on the coarse grid and corrected by the Kalman filter.

This behavior highlights how observations are much more significant at the beginning of the simulation, when the knowledge associated with the model is lower (because there is less information about the past history of the model evolution), and experimental data somehow compensate this lack of information. As the simulation goes on, the model evolves as well, and more and more information about its past history are available. Therefore, the importance of observations decreases in time, until a stationary value is reached. In Figure 6, which represents the time evolution of the maximum value of the normalized misfit, computed as the greatest absolute point-wise difference between observation and time solution, it can be seen how, when the gain reaches a stationary value, the misfit value presents a plateau. This implies that the relevance of observations is very low (but not zero), and that convergence has been reached. As a further figure of merit, Table 2 compares the overall values of the $\chi^{2}$ over all time steps of the solution computed with the standard algorithm and the one computed with the integrated one. As expected, the $\chi^{2}$ for the integrated algorithm is much lower than the one for the standard algorithm, signaling a better fit with the model.

**Figure 7:**
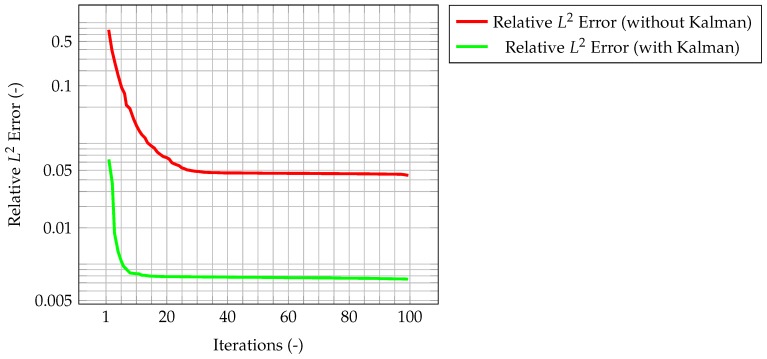
Time evolution of the maximum normalized $L^{2}$ error between observation and CFD solution. The red line is computed as the error between the reference solution computed on the fine grid, and the one evaluated on the coarser grid, without the correction by the filter. In the case of the green line, the CFD solution on the coarser grid has been corrected by the Kalman filter.

As an evaluation of the effect of the Kalman filter, the misfit evaluated for the corrected velocity on a coarse grid is compared to the one evaluated for a non-corrected velocity on the same grid. In both cases, convergence is reached fairly quickly, however for the former the final, stationary value is one order of magnitude lower than the latter case, highlighting the performance improvement of the CFD algorithm due to the implementation of a Kalman filter correction, and without the need of a mesh refinement. The same behavior can be seen in Figure 7, when computing the normalized $L^{2}$ error. These plots show that the time solution computed with the developed algorithm is quite close both to the CFD one and to the observation, and that the performance of the filter on the coarse grid is much better than the one for the segregated method on the same mesh.

In terms of computational times, the use of the integrated algorithm causes an increase of the time needed to perform a single time iteration, and the convergence speed is lower than the segregated method only. However, given the same grid the results obtained with the filter are more accurate than the one obtained with the segregated method only. The addition of the filter to the CFD solver causes an increase of roughly 20% in terms of computational time with respect to the case without it (considering the same coarse grid), while providing higher accuracy. On the other hand, this increase is much lower than the one caused by mesh refinement, which is about ten times as much, as shown in Table 3.

#### 4.2.2. Kalman-PIMPLE Algorithm

In the second test, the segregated PIMPLE algorithm is coupled with the standard Kalman filter. The simulation is again performed until 100 s, with an initial time step of $5\cdot 10^{-3}$ s. With respect to the PISO method shown above, PIMPLE is characterized by an embedded time-adaptivity feature, which depends on the maximum value of an a-dimensional quantity known as the Courant number, defined as follows:

(48) $$Co=\frac{u\delta t}{\delta x}$$

At every iteration, a new time step is computed, such that the maximum Courant number remains below a certain threshold. This feature of the PIMPLE algorithm must be stressed because the uncertainty associated to the model is related to the time step. Since this uncertainty ultimately affects the augmented prediction, the influence of time adaptivity on the performance of the integrated algorithm must be checked. It is important to point out that the PISO and PIMPLE algorithms solve the same equations, and that the main difference between the two is that the latter uses an inner predictor–corrector loop for the momentum and pressure equations, solving them more times within the same iteration to speed up convergence and accuracy.

**Figure 8:**
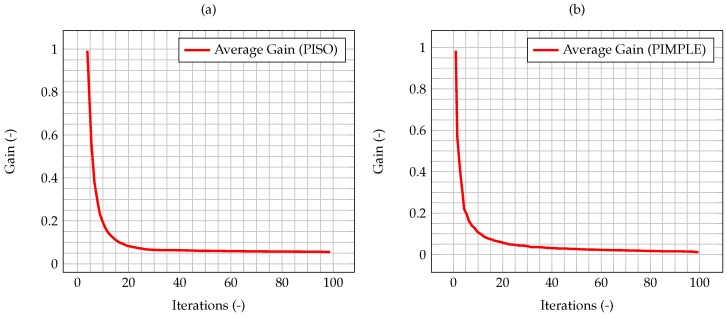
Time evolution of the average Kalman gain for the PISO algorithm with fixed time step equal to 0.005 s (**a**) and the PIMPLE algorithm (**b**) with time adaptivity and initial time step equal to 0.005 s.

In Figure 8, the time evolution of the average Kalman gain for the algorithm with time adaptivity is shown. As can be seen, the behavior of the gain is exactly the same as the one shown by the PISO algorithm, despite the varying time step.

**Figure 9:**
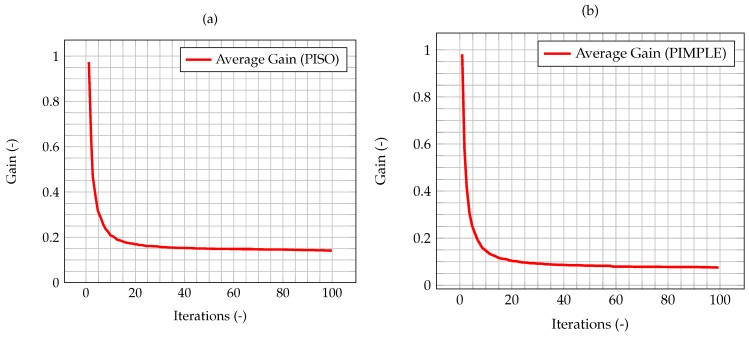
Time evolution of the average Kalman gain for the PISO algorithm (**a**) and the PIMPLE algorithm (**b**) with time adaptivity and initial time step equal to 0.01 s.

**Figure 10:**
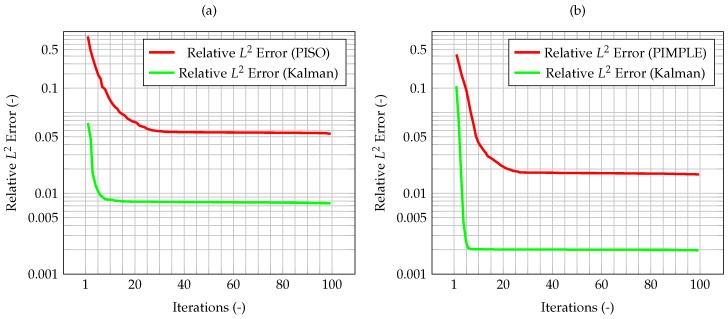
Time evolution of the normalized $L^{2}$ error for the PISO (**a**) and the PIMPLE algorithm (**b**) with time adaptivity and initial time step equal to 0.01 s. In both cases, the red lines represent the difference between the reference solution and the one computed on the coarser grid without correction, whereas in the green ones the coarser solution is corrected by the filter.

As shown by Equation (40), the uncertainty associated to the model has been modeled as dependant from the time step. Therefore, the differences between the two algorithms will be more significant as the error on the numerical model increases. To highlight the influence of the time step and the time-adaptivity feature of the PIMPLE method, an higher initial δt, equal to 0.01 s, is used, enough to show the difference on the performance of the Kalman filter between the PISO and PIMPLE algorithms. Both the average gain (Figure 9) and the normalized $L^{2}$ error (Figure 10) are lower in the case of PIMPLE, as expected since the adaptive time step is used. It is thus expected that an increase of the time step will lead to worse performance of the filter, in terms of higher average gain and higher normalized $L^{2}$ error.

The present analysis leads to the conclusion that the Kalman gain decreases monotonically with both algorithms. Its performance depends on the specific application the filter is studied on, and in particular whether the local value of the Courant number (Equation (48)) is such that it causes an increase or decrease of the time step. The influence of the time step is strictly related on the error associated to the model, through the term **Q**. When the model uncertainties are lower, the influence of the time step on the filter performance is lower. In the same vein, when the error associated with the model is not negligible with respect to the one associated with the observation, the model prediction is always corrected by the filter with the experimental data, and thus the gain does not go to zero even when a steady-state is reached.

**Figure 11:**
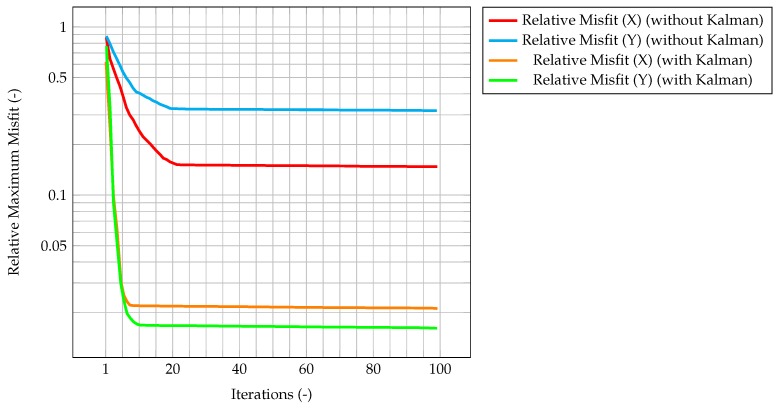
Time evolution of the maximum normalized misfit between observation and CFD solution (with and without correction by the filter).

In Figure 11, the time evolution of the maximum value of the normalized misfit, computed as the greatest absolute point-wise difference between observation and time solution, is described. It can be seen how this value quickly decreases to $10^{-1}$, comparable with the one obtained by the PISO algorithm. Despite the higher δt, the misfit is quite similar, meaning that, in this particular test study, the PIMPLE algorithm is performing better than the PISO one.

Table 4 compares the overall values of the $\chi^{2}$ over all time steps of the solution computed with the standard algorithm and the one computed with the integrated one. Again, the $\chi^{2}$ for the integrated algorithm is much lower than the one for the standard algorithm, signaling a better fit with the model even with respect to the PIMPLE algorithm.

In terms of computational times, the time required by the PIMPLE algorithm to perform a single iteration is roughly the same as the one needed by the PISO method, however the convergence speed for the fine mesh is much lower. The advantage of using the integrated method is much more evident in this case than in the previous one. Indeed, at the expense of a slight increase of the convergence time, the integrated algorithm gives results comparable to the ones obtained using a fine mesh. The increase in time needed by the filter remains very small compared to the one needed by mesh refinement. These results are summarized in Table 5.

#### 4.2.3. Kalman-Temperature Algorithm

**Figure 12:**
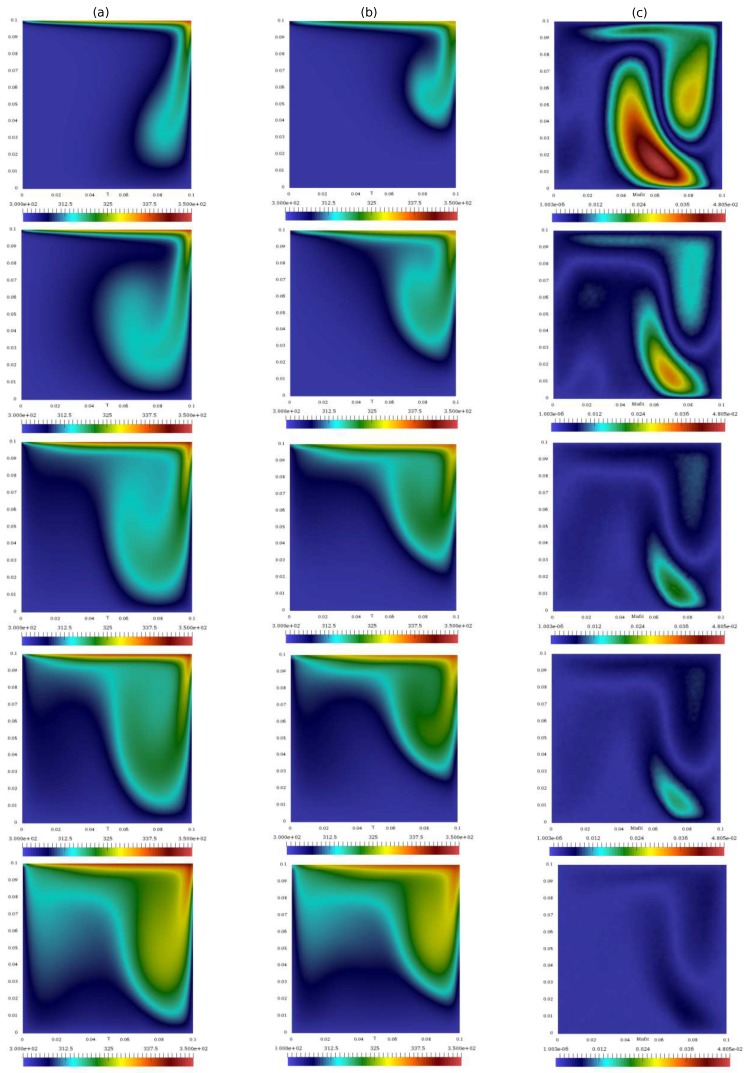
Temperature profile within the domain of: the coupled algorithm with Kalman filter (**a**); the standard PISO algorithm (**b**); and the difference between the time solutions of the filter and of the standard algorithm computed on the finest mesh (**c**).

In this test, the temperature equation under the Boussinesq approximation is added to the segregated algorithm. When dealing with heat transfer, the coupling between velocity and temperature must be considered along with the one between velocity and pressure. However, the Kalman filter does not take into account this relationship, and the two quantities are predicted separately. In the following, only temperature measurements are adopted for the filter to check whether the algorithm is able to improve the accuracy of both the temperature prediction and the velocity prediction. This is of particular interest because, as mentioned above, is not straightforward to obtain experimental data on velocity. Again, the simulation is performed until 100 s with a constant time step of $5\cdot 10^{-3}$ s. Figure 12 shows the temperature of the standard algorithm and of the integrated algorithm (evaluated on the same grid) at different times, along with the difference between the integrated and the reference one computed on the fine grid. Again, the behavior of the misfit in the domain can be used for a posteriori optimization of the sensor’s location.

**Figure 13:**
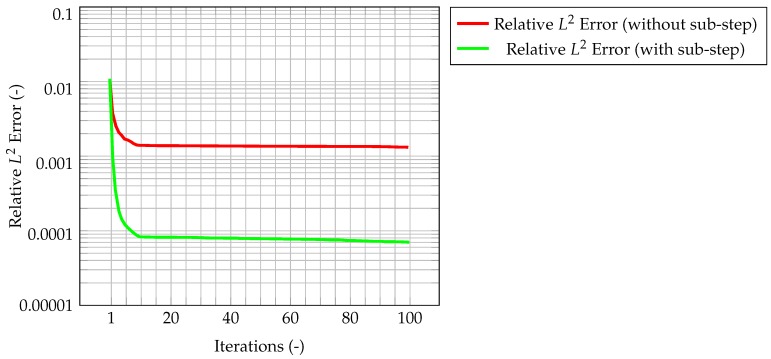
Time evolution of the normalized $L^{2}$ error for the mass flux. This error represents the difference between the reference mass flux computed on the fine grid, and the ones computed on the coarser ones with the correction by the filter, respectively, without (red) and with (green) the sub-stepping correction in the algorithm.

Now, the quantity that couples temperature and velocity is the mass flux. First computed from the latter, it is used in the prediction of the former. Therefore, it is likely that, if the mass flux computed from the corrected velocity remains consistent with the one evaluated from the segregated algorithm, the integrated method will be able to correctly predict the state of the system. For this reason, the normalized $L^{2}$ error for the mass flux is computed and shown in Figure 13. Clearly, the mass conservative sub-step within the algorithm is necessary to have a consistent mass flux. Indeed, the normalized $L^{2}$ error for the flux, as computed by the algorithm with mass conservative sub-step, is one order of magnitude lower than the one computed by the algorithm without sub-step. This also guarantees the solution to be mass conservative.

**Figure 14:**
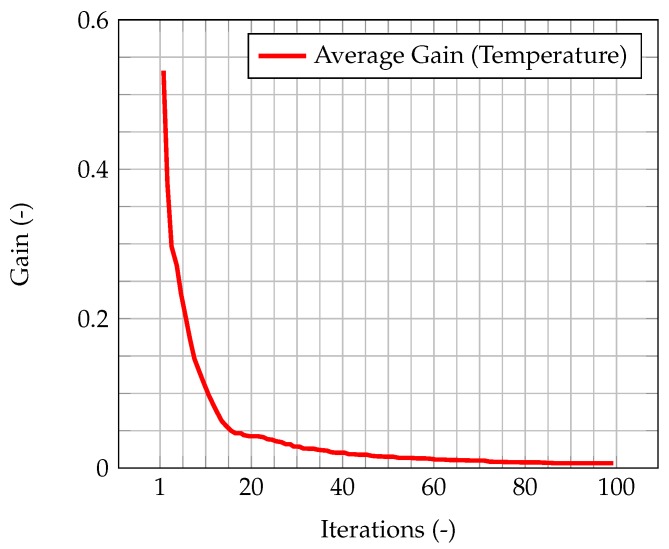
Time evolution of the average Kalman gain for temperature, highlighting its exponential-like behavior and the stationary value reached at convergence.

The results for the coupled case are now presented. Figure 14 shows the time evolution of the average Kalman gain for temperature. As seen in the previous cases, this behavior highlights how observations are much more significant at the beginning of the simulation, when the knowledge associated with the model is lower (because there is less information about the past history of the model evolution), and experimental data somehow compensate this lack of information. As the simulation goes on, the model evolves as well, and more and more information about its past history is available. Therefore, the importance of observations decreases in time, until a stationary value is reached.

**Figure 15:**
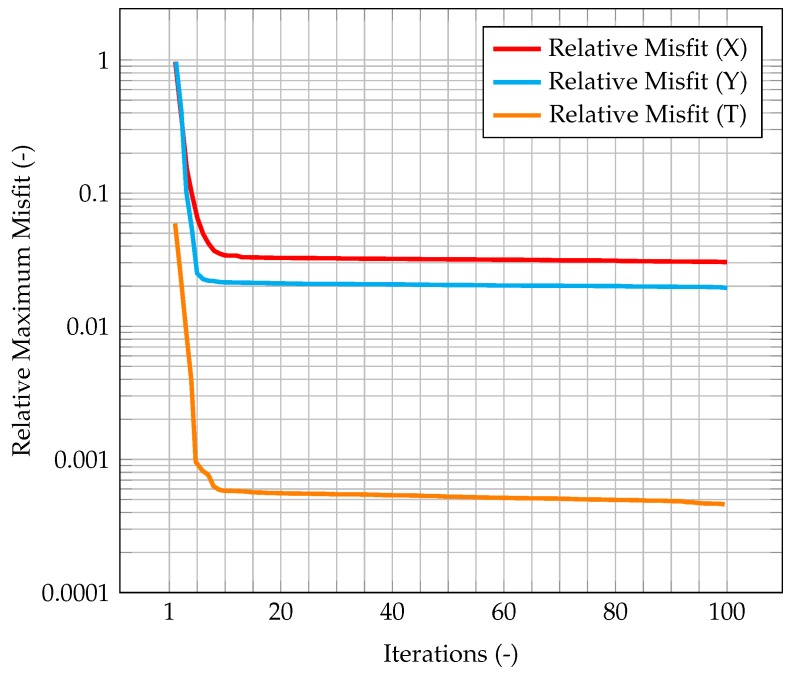
Time evolution of the maximum normalized misfit between observation and solution. Note that the velocity is not actually corrected by the filter; rather, temperature is corrected, and this *augmented* value is used in the prediction of the velocity field.

**Figure 16:**
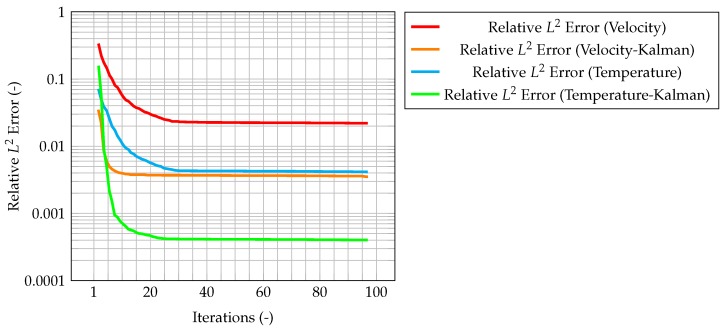
Normalized $L^{2}$ error for velocity and temperature. In the figures, the red and blue line represent the error evaluated between the reference solution and the one evaluated on a coarser grid without the correction provided by the filter, respectively, for velocity and temperature. In the case of the green line, temperature is now corrected by the Kalman filler, and thus velocity is computed starting from *improved* temperature values (however, the velocity itself is not corrected).

In Figure 15, the maximum value of the normalized misfit, computed as the greatest absolute point-wise difference between observation and time solution, is shown both for velocity and temperature. It is worth reminding how only temperature observations are available, meaning that only the temperature prediction is augmented by the Kalman filter, and indeed the maximum misfit for temperature quickly decreases below $10^{-3}$. However, the most significant result is that also the maximum misfit for velocity decreases, despite observations for this quantity not being available. This implies that also the velocity prediction is being augmented by the presence of temperature observations only, thus proving the ability of the algorithm to provide an accurate and improved estimation of both quantities of interest even without experimental data on velocity. This can be explained by the fact that the velocity field is now computed considering the *augmented* temperature field. The same behavior can be seen in Figure 16, when computing the normalized $L^{2}$ error. These plots show that the time solution computed with the developed algorithm is quite close both to the CFD one and to the observations.

Table 6 compares the overall values of the $\chi^{2}$ over all time steps of the solution computed with the standard algorithm and the one computed with the integrated one, for both velocity and temperature. As expected, for both quantities, the $\chi^{2}$ for the integrated algorithm is much lower than the one for the standard algorithm, signaling a better fit with the model.

In terms of computational times, the use of the integrated algorithm causes an increase of the time needed to perform a single time iteration, and the convergence speed is lower than the segregated method only. However, given the same grid, the results obtained with the filter are more accurate than the one obtained with the segregated method only. The addition of the filter to the CFD solver causes an increase of roughly 30% in terms of computational time with respect to the case without it (considering the same coarse grid), while providing higher accuracy. On the other hand, this increase is much lower than the one caused by mesh refinement, which is about ten times as much, as shown in Table 7.

## 5. Conclusions

The present research work describes the development of a new solver for incompressible flow based on the integration between the segregated methods and the Kalman filter. Following the work found in [16], the aim of this approach is to reduce the uncertainties associated with the numerical simulation by the combination of experimental data within the computational framework. This integration leads to the evaluation of an augmented flow state prediction, accounting for both the level of confidence in the model and in the observation. Through the use of the segregated methods, which perform a linearization of the system of equations that characterizes non-isothermal incompressible flows, mass conservation of the augmented state is guaranteed.

As a preliminary test, the developed method was studied against the classic benchmark of the 2D lid-driven cavity. Three different cases, of increasing complexity, were considered. First, the Kalman filter was integrated with the standard PISO method. Secondly, the integration of the filter was extended to the PIMPLE algorithm, taking into account its time-adaptivity feature. Lastly, the algorithm was further expanded to allow the integration of temperature observations. In this case, despite the presence of temperature experimental data only, it was shown how the algorithm is able to improve the prediction of both velocity and temperature. In all the above cases, to test the capabilities of the new algorithm, synthetic measurements for the two quantities of interest were provided on a limited number of grid elements.

Overall, the developed method shows better performances than the standard segregated approach (with respect to the same numerical grid), at the expense of an increase of computational time roughly equal to 15%. The obtained results also highlight how the integration of the filter with the PIMPLE algorithm offers some advantages with respect to the PISO one, while requiring additional computational time. Future works will be devoted to the extension of this technique, for example by extending the algorithm to deal with compressible fluids and to model heat transfer without the Boussinesq approximation. The development of a posteriori model for the generation of the probability distribution of the measurement noise (in the present work, a Gaussian distribution was considered as an a priori model) in the case of real observation data is also a point of interest. In addition, the combination of the developed model with a reduced order method is currently under study [27,28,29].

All the examples and figures are available online at https://figshare.com/articles/An_implementation_of_the_mass_conservative_Kalman_Filter_for_computational_thermo-fluid_dynamics_/7177004 [30].

## Figures and Tables

**Table 1:** Summary of the algorithms and cases.

Method	Kalman Correction (Velocity)	Kalman Correction (Temperature)
PISO + KF	Yes	No
PIMPLE + KF	Yes	No
Temperature + KF	Yes	No

**Table 2:** Comparison between the $\chi^{2}$ for the segregated method and the integrated algorithm.

Segregated	Integrated
0.145	0.065

**Table 3:** Comparison between the run times for the segregated method and the integrated algorithm.

Test Case	Iteration Time	Convergence Time	Convergence Iterations
PISO + KF	2.60 s	29.88 s	290
PISO	2.09 s	19.99 s	250
PISO (Fine mesh)	20.87 s	198.87 s	2495

**Table 4:** Comparison between the $\chi^{2}$ for the segregated method and the integrated algorithm.

PISO	PIMPLE	Integrated
0.145	0.096	0.065

**Table 5:** Comparison between the run times for the segregated method and the integrated algorithm.

Test Case	Iteration Time	Convergence Time	Convergence Iterations
PIMPLE + KF	3.01 s	26.54 s	1256
PIMPLE	2.28 s	17.74 s	1104
PIMPLE (Fine mesh)	109.03 s	2679.58 s	6750

**Table 6:** Comparison between the $\chi^{2}$ for the segregated method and the integrated algorithm.

Quantity	Segregated	Integrated
Velocity	0.185	0.085
Temperature	25.65	18.43

**Table 7:** Comparison between the run times for the segregated method and the integrated algorithm.

Test Case	Iteration Time	Convergence Time	Convergence Iterations
Temperature+Kalman	3.84 s	50.43 s	3171
Temperature	2.69 s	37.66 s	2799
Temperature (Fine mesh)	148.53 s	412.57 s	4000

## Supplementary Materials

Supplementary File 1
