# Supplementary material for: A Mass Conservative Kalman Filter Algorithm for Computational Thermo-Fluid Dynamics
Source: Materials (Basel). 2018 Nov 8;11(11):2222. doi: 10.3390/ma11112222 (PMC6267179; doi:10.3390/ma11112222)
Supplement: Supplementary File 1 [file materials-11-02222-s001.pdf]

A Mass Conservative Kalman Filter Algorithm for Computational Thermo-Fluid Dynamics

Supplementary Materials could be found by the following link:

https://figshare.com/articles/An_implementation_of_the_mass_conservative_Kalman_Filter_for_computational_thermo-fluid_dynamics_/7177004.
